# Mismatch repair gene *MSH6* correlates with the prognosis, immune status and immune checkpoint inhibitors response of endometrial cancer

**DOI:** 10.3389/fimmu.2024.1302797

**Published:** 2024-02-08

**Authors:** Lin-Zhi Zhou, Hong-Qi Xiao, Jie Chen

**Affiliations:** ^1^ Department of Gynecological Oncology, Harbin Medical University Cancer Hospital, Harbin, China; ^2^ Department of General Surgery, The Second Affiliated Hospital of Harbin Medical University, Harbin, China

**Keywords:** endometrial cancer, mismatch repair, immune infiltration, immune checkpoint, immune checkpoint inhibiter, prognosis, MSH6

## Abstract

**Objective:**

Many patients treated with immune checkpoint inhibitors (ICIs) developed primary or secondary drug resistance for unknown reasons. This study investigates whether mismatch repair (MMR) genes are responsible for this therapeutic restriction.

**Methods:**

We obtained the transcriptional, clinical and single nucleotide polymorphism data for endometrial cancer (EC) from The Cancer Genome Atlas and the immunophenoscore data of EC from The Cancer Immunome Atlas, then analyzed in R to evaluate the relationship between MMR genes and clinicopathological features, prognosis, immune infiltration, immune checkpoint expression and responsiveness to ICIs in EC. We used differentially expressed genes in the *MSH6* high and low expression groups to conduct GO and KEGG analyses to explore the impact of *MSH6* on the biological functions of EC. Finally, we verified the bioinformatics results with *in vitro* experiments.

**Results:**

Our analyses showed that compared with the high *MSH6* expression group, the low *MSH6* expression group had better survival outcomes and less aggressive clinicopathological features. In the multivariate Cox analysis, *MSH6* was the only independent risk factor that could predict the prognosis of EC. Besides, the low *MSH6* expression group also had a higher immune score, more active immune infiltration and higher immune checkpoint expression, resulting in better responsiveness to ICIs treatment, consistent with the enrichment of GO terms and KEGG pathways related to immune response in this group. Meanwhile, the GO and KEGG enrichment results of the *MSH6* high expression group were associated with cell cycle, DNA damage repair and tumorigenesis. To exclude the influence of *MSH6* mutations, we performed the previous analyses on the *MSH6* wild-type tumor samples and obtained consistent results. *In vitro* experiments also confirmed that after knocking down *MSH6* in endometrial cancer cells, their proliferation, migration and invasion abilities were weakened, while the expression levels of PD-L1 and PD-L2 were elevated. In comparison, overexpression of *MSH6* showed an opposite trend.

**Conclusion:**

Reduced *MSH6* expression could serve as a potential biomarker for predicting better prognosis, active immune status, higher immune checkpoint expression level and better responsiveness to ICIs treatment in EC. *MSH6* may become a potential target for treating solid tumors.

## 1 Introduction

In recent years, due to the extension of life expectancy and the increase in the obesity rate, the morbidity and mortality of endometrial cancer (EC) have continued to rise and show a younger trend (1, 2). EC is typically categorized into type I and type II according to clinical, endocrine and epidemiological features, or into endometrioid, serous and clear cell carcinoma based on histopathological characteristics (3). Many cases have demonstrated that both categorization schemes can accurately identify the nature and prognosis of most tumors. However, in some cases, the tumor morphology is vague, and the characteristics overlap, making it challenging to categorize accurately, resulting in overtreatment or insufficient treatment. In 2013, the American Cancer Genome Atlas (TCGA) divided ECs into four molecular subtypes based on multi-omics features and gradually optimized them: *POLE* ultra-mutated, microsatellite instability-high (MSI-H)/mismatch repair deficient (dMMR), *p53* abnormal and no specific molecular profile (4, 5). This classification is significant in predicting patients’ prognosis and recurrence risk and can provide individualized diagnosis and treatment strategies.

Among the four molecular subtypes, the MSI-H/dMMR subtype accounts for approximately 30% of all primary ECs and 13% to 30% of all recurrent ECs (6), which is caused by mutations (germline pathogenic variants or double somatic pathogenic variants) or epigenetic changes in four MMR genes (*MLH1*, *MSH2*, *MSH6*, *PMS2*) *(* 7, 8). This subtype is characterized by high tumor mutational burden (TMB), increased tumor-infiltrating lymphocytes and upregulated expression of immune checkpoints, making it an ideal target for immune checkpoint inhibitors (ICIs) (6, 9, 10). In May 2017, based on the findings of many clinical trials (11–13), the US Food and Drug Administration (FDA) accelerated the approval of pembrolizumab for the treatment of refractory adult and pediatric MSI-H/dMMR solid tumors, including EC (14). In 2021, this classification system was formally incorporated into the NCCN guidelines for uterine neoplasms, with precise detection and treatment protocols developed (15). By blocking programmed cell death 1 ligand (PD-L1) on tumor cells and programmed cell death receptor 1 (PD-1) or cytotoxic T lymphocyte antigen-4 (CTLA-4) on T cells, ICIs can inhibit their immunosuppressive interactions, reactivate the exhausted immune cells in the tumor microenvironment (TME), and restore the antitumor effect of effector T cells (16, 17).

ICIs have revolutionized cancer treatment, but the response of patients with MSI-H/dMMR tumors (different origins or the same origin) varies greatly. Some patients may be susceptible to ICIs and have responsiveness durably, while nearly half of the patients fail to benefit from them for unknown reasons (18). We want to explore whether different MMR gene defects are to blame for this treatment restriction from the perspective of the MMR gene itself and whether we could develop drugs that specifically target these different MMR genes to improve patients’ responsiveness to ICIs in the future. Besides, effective biomarkers are required to guide patient selection. In addition to MSI-H/dMMR, other commonly used markers for predicting reactivity include PD-L1, tumor-infiltrating lymphocytes and TMB, but these markers are not entirely reliable (19, 20). To address these issues, we investigated the connections between MMR genes and clinicopathological characteristics, prognosis, immune infiltration, immune checkpoint expression and response to ICIs in EC at the gene level by bioinformatics analysis, and the results were then confirmed by *in vitro* experiments.

## 2 Materials and methods

### 2.1 Bioinformatics analysis

#### 2.1.1 Data acquisition and processing

We obtained EC’s transcriptional and clinical data from TCGA (https://portal.gdc.cancer.gov/) and the survival information on pan-cancer from the UCSC XENA database (https://xenabrowser.net/datapages/) (21). Perl scripts (https://www.perl.com/) were used to merge and preprocess the raw data to extract the gene expression matrix and clinical information. A total of 35 normal samples and 552 tumor samples were collected.

#### 2.1.2 Survival analysis

The survival information of TCGA-UCEC was screened, including survival status, overall survival (OS), progression-free survival (PFS), disease-specific survival (DSS) and disease-free survival (DFS). Then, they were merged with the MMR gene expression data in tumor samples and divided into high and low expression groups according to the medium FPKM value of MMR gene. “survival” (https://cran.r-project.org/web/packages/survival/index.html) and “survminer” packages (https://cran.r-project.org/web/packages/survminer/index.html) were invoked in R Version 4.1.3 (https://www.r-project.org/) to analyze the survival difference between the high and low expression groups, “timeROC” package (https://cran.r-project.org/web/packages/timeROC/index.html) was used to assess the predictive accuracy of MMR genes (22).

#### 2.1.3 The correlation of MMR gene with clinicopathological features and prognosis

We used the “limma” package (https://bioconductor.org/packages/release/bioc/html/limma.html) in R to analyze whether there were differences in clinicopathological characters between the high and low expression groups of MMR genes. The “ggpubr” package (https://cran.r-project.org/web/packages/ggpubr/index.html) was used to analyze whether there were differences in MMR gene expression among different clinicopathological features. The “survival” package was used for univariate and multivariate Cox regression analyses to determine the independent predictors related to prognosis.

#### 2.1.4 The correlation of MMR gene with tumor immune microenvironment and immune checkpoint inhibitor response

The “ESTIMATE” algorithm was performed to calculate the immune, stromal and ESTIMATE scores for each tumor sample (23). The Wilcoxon test was used to analyze whether there were significant differences in the three scores between the MMR gene high and low expression groups (23). The “CIBERSORT” package (https://cibersortx.stanford.edu/) was called in R to analyze whether there were differences in immune cell infiltration levels between the two groups (23). Spearman’s correlation test was used to analyze the correlation of the MMR gene with immune cell infiltration and immune checkpoint related gene expression (23).

The immunophenoscore (IPS) data of EC patients was downloaded from the Cancer Immunome Atlas (TCIA) (https://tcia.at/home). Then, it was merged with the MMR gene expression data in tumor samples to compare whether there were differences in multiple IPS scores between high and low MMR gene expression groups (24).

#### 2.1.5 Repeat analysis in *MSH6* wild-type tumor samples after excluding those with *MSH6* mutations

We downloaded the single nucleotide polymorphism data of UCEC from TCGA and obtained 432 *MSH6* wild-type tumor samples and 75 *MSH6* mutant tumor samples. R was used to screen the expression data of *MSH6* and immune checkpoint related genes, survival information, immune, stromal and ESTIMATE scores, 22 types of immune cell infiltration data and IPS data of all *MSH6* wild-type tumor samples. We also divided the *MSH6* wild-type tumor samples into high and low *MSH6* expression groups according to the median FPKM value of *MSH6* and analyzed whether there were differences in survival, immune score, immune infiltration, immune checkpoint expression and ICIs treatment responsiveness between the two groups using the same methods as before.

#### 2.1.6 Functional enrichment analyses

We used the “limma” package in R to obtain the differentially expressed genes (DEGs) between the high and low expression groups of *MSH6*, with | log_2_ fold change (FC) |>1 and adjusted *p*<0.05 as filtering conditions. The “clusterProfiler” package (https://bioconductor.org/packages/release/bioc/html/clusterProfiler.html) was used in R to conduct Gene Ontology (GO) and Kyoto Encyclopaedia Genes and Genomes (KEGG) analyses on the DEGs that were upregulated in *MSH6* high and low expression groups. Results with a false positive rate (FDR) q value<0.05 were deemed significant and were subsequently visualized using the “ggplot2” (https://cran.r-project.org/web/packages/ggplot2/index.html) and “enrichplot” (https://bioconductor.org/packages/release/bioc/html/enrichplot.html) packages.

### 2.2 *In vitro* assay

#### 2.2.1 Cell culture and transfection

Endometrial cancer cells Ishikawa and HEC-1B and 293T cells were purchased from Shanghai Fuheng Biotechnology Co., Ltd. All cells were cultured in Dulbecco’s modified Eagle’s medium (DMEM) containing 10% fetal bovine serum (FBS) and 1% penicillin/streptomycin at 37 °C with 5% CO2. We named the lentiviral vector that can downregulate the expression of *MSH6* and its control vector as shMSH6 and shNC, respectively, and the vector that can upregulate the expression of *MSH6* and the empty vector as OE-MSH6 and Vector, respectively, all of which were purchased from Wuhan Weizhen Biological Company (China). The vector plasmid (10 µg), the helper plasmid psPAX2 (5 µg) and the helper plasmid pMD2G (5 µg) were transfected into 293T cells with Neofect® DNA transfection reagent (Beijing Neofect Biotech Co., Ltd.) at a ratio of 2:1:1. We collected the supernatant containing virus particles 48 hours after transfection and used it to infect Ishikawa and HEC-1B cells after centrifugation and filtration. Target cells were screened with a complete medium containing 2 µg/mL puromycin for 7-14 days after infection with viral particles for 48 hours to obtain stably transfected cell lines. The transfection results were verified by western blotting (WB) and reverse transcription-quantitative polymerase chain reaction (RT-qPCR) analysis. The sequence of shMSH6 was CCG GTT CTG ACA AAG GTG GTA AAT TCT CGA GAA TTT ACC ACC TTT GTC AGA ATT TTT G; The sequence of shNC was TTC TCC GAA CGT GTC ACG TTT CAA GAG AAC GTG ACA CGT TCG GAG AAT TTT TT; The sequence of OE-MSH6 was referred to NM_000179.

#### 2.2.2 Western blotting

Cells were lysed with RIPA buffer containing protease inhibitors, and protein concentration was then detected using the BCA Protein Assay Kit (Beyotime, China). According to the manufacturer’s recommendations, 50 µg protein samples per well were separated by 10% sodium dodecyl sulfate-polyacrylamide gel electrophoresis (SDS-PAGE, Beyotime, China) and transferred the protein on the gel to the PVDF membrane, blocked the membrane with 2.5% skimmed milk for 1 hour at room temperature, and then incubated with the primary antibody against GAPDH, MSH6, PD-L1 and PD-L2 at 4°C overnight, finally incubated with the corresponding second antibody. After washing with PBST 3 times, the bands were visualized with an ECL detection reagent (meilunbio^®^, China). Quantitative analysis of protein expression was performed using ImageJ.

#### 2.2.3 RT-qPCR

According to the manufacturer’s recommendation, total RNA was extracted from cells using the TRIzol regent. Using a PrimeScript™ RT reagent Kit with gDNA Eraser (Perfect Real Time) (TAKARA, RR047Q, Japan) to reverse transcribed 1 µg RNA into cDNA, followed by quantitative real-time PCR using a TB Green® Premix Ex Taq™ II (Tli RNaseH Plus) (TAKARA, RR820A, Japan). The primers used in the experiment were synthesized by Beijing Ruibo Xingke Biotechnology Co., Ltd. in China, including GAPDH forward 5’-GGTGTGAACCATGAGAAGTATGA-3’and reverse 5’-GAGTCCTTCCACGATACCAAAG-3’; MSH6 forward 5’-GGCTCGAAAGACTGGACTTATT-3’and reverse 5’-CCAGGAGGCTCTGTTCATTT-3’; CD274 forward 5’-GCTGAATTGGTCATCCCAGAA-3’and reverse 5’-CAGTGCTACACCAAGGCATAA-3’; PDCD1LG2 forward 5’-CATGTGAACCTTGGAGCAATAAC-3’and reverse 5’-CCTCACTTGGACTTGAGGTATG-3’.

#### 2.2.4 Cell proliferation and clone formation assay

After constructing endometrial cancer cell lines with stable knockdown and overexpression of *MSH6*, changes in cell proliferation activity were detected by cell proliferation and clone formation assay.

In the cell proliferation assay, the cells were seeded into 96-well plates at a ratio of 2,000 cells per well. Subsequently, 10 µl of CCK-8 reagent (Beyotime, China) was added to each well at 24 hours, 48 hours, 72 hours, 96 hours and 120 hours, respectively, according to the recommendations of the reagent manufacturer. The absorbance at 450 nm was measured with a microplate reader after incubation at 37°C for 2 hours.

In the clone formation assay, 2,000, 1,000 and 500 cells were seeded into 6-well plates for culture, and fresh complete medium was regularly replaced for 7-14 days until visible colonies were observed. The cell colonies were fixed with methanol for 30 minutes, stained with 2.5% crystal violet (Solebol, China) for 30 minutes, washed, dried, photographed and counted the colonies formed. Cloning efficiency (%) = (number of colonies formed/number of cells inoculated) × 100%.

#### 2.2.5 Wound healing assay and transwell

The cells were spread into the 6-well plate one day in advance to ensure the density was above 90% the next day. The wound was made with a 200 µl yellow pipette tip perpendicular to the bottom of the plate. After the medium was discarded, the wound was cleaned twice with PBS to fully wash the cells in the scratch gap, and 2ml serum-free medium was added to each well. The inverted microscope was used to take photos at 0 hours and 48 hours after wound formation; the scratch area was measured at different time points with Image J (A0, A48), and the cell migration rate was calculated as follows: (A0-A48)/A0 x 100%.

The chamber (24-well, 8um pore size) and Matrigel used for the Transwell assay were purchased from Corning (United States). According to the manufacturer’s recommendation, 600 µL DMEM containing 20% FBS was added to the lower chamber, and 200 µL cell suspension with a density of 4X10^5 cells/mL was added to the upper chamber. After incubation for 36 hours, the upper chamber was taken out, the cells that migrated to the lower chamber were fixed with methanol for 30 minutes, stained with 2.5% crystal violet for 30 minutes, cleaned with deionized water and dried. Five fields were randomly photographed under an inverted microscope with a magnification of 100X for counting, and the average value was taken as the number of cells that passed through. In the invasion assay, the Matrigel was thawed at 4°C overnight in advance. When using, diluted the Matrigel 8 times with DMEM and added 50 µl to the upper chamber; the entire process was performed on ice. The chamber coated with Matrigel can be used as before mentioned after incubation at 37°C and 5% CO2 for 3 hours.

### 2.3 Statistical analysis

Statistics for all bioinformatic analyses were performed in R Version 4.1.3. The Wilcoxon test was used when comparing two groups, and the Kruskal-Wallis test was used when comparing three or more groups. Correlation analyses between two variables were performed using the Spearman test. In the *in vitro* assay, all experiments were repeated three times, and Student’s *t*-test was performed using GraphPad Prism 9.0.0 to evaluate whether there were statistical differences between two independent groups. Unless otherwise mentioned, *P*<0.05 was considered statistically significant in all analyses.

## 3 Results

### 3.1 Prognostic value of MMR genes in EC

**Figure 1:**
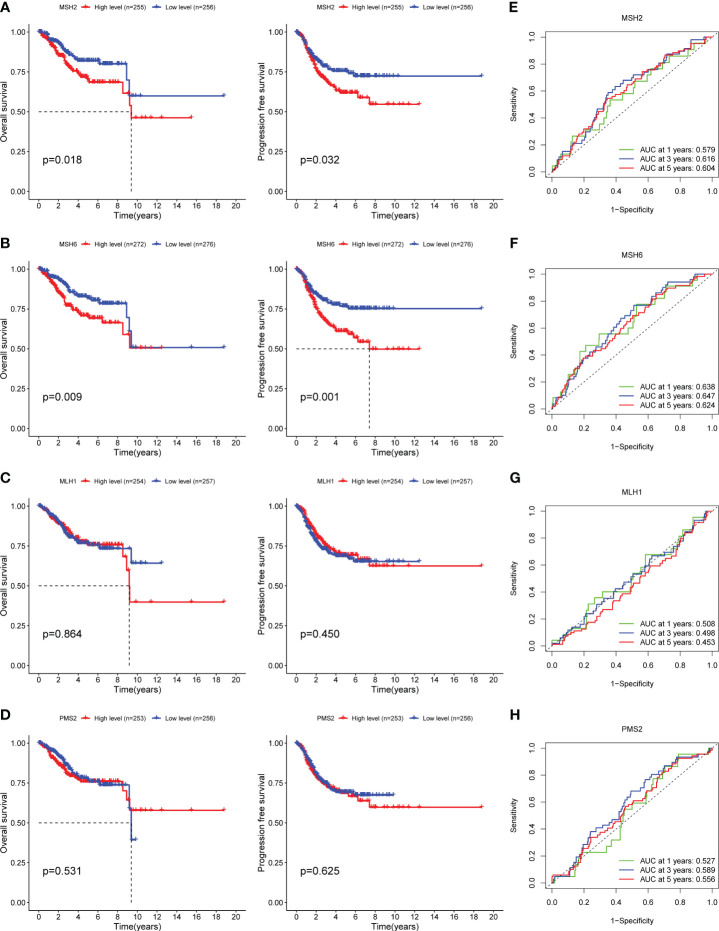
*MSH2* and *MSH6*, but not *MLH1* and *PMS2*, related to the OS and PFS of EC patients. The OS and PFS curves of the low expression groups of *MSH2* **(A)** and *MSH6* **(B)** were significantly higher than those of their high expression groups, while there was no significant difference between the high and low expression groups of *MLH1* **(C)** and *PMS2* **(D)**. The area under the ROC curve of *MSH2* **(E)** and *MSH6* **(F)** was higher than that of *MLH1* **(G)** and *PMSH2* **(H)**. OS, Overall Survival; PFS, Progress Free Survival; EC, endometrial cancer.

MMR genes maintain genomic stability and inhibit tumor formation by preventing mutation accumulation and mediating apoptotic responses of DNA damage, while mutations in MMR genes cause hereditary non-polyposis colorectal cancer, and MMR defects are associated with the formation of multiple sporadic tumors (25, 26). Multiple studies have confirmed that the overexpression of MMR proteins is associated with adverse survival outcomes in a variety of tumors, including prostate cancer (27), oral squamous cell carcinoma (28), melanoma (29), etc. To determine whether MMR genes can predict the prognosis of EC patients or not, we divided EC samples into high and low expression groups based on the median FPKM value of MMR genes and found that the survival outcomes of the *MSH2* and *MSH6* low expression groups were significantly better than their high expression groups (*MSH2*: OS, *p*=0.018, **Figure 1A**; PFS, *p*=0.032, **Figure 1A**; DSS, *p*=0.033, **Supplementary Figure S1A**; DFS, *p*=0.103, **Supplementary Figure S1A**; *MSH6*: OS, *p*=0.009, **Figure 1B**; PFS, *p*=0.001, **Figure 1B**; DSS, *p*<0.001, **Supplementary Figure S1B**; DFS, *p*=0.015, **Supplementary Figure S1B**);, while there was no significant difference between the high and low expression groups of *MLH1* and *PMS2* (*MLH1*: OS, *p*=0.864, **Figure 1C**; PFS, *p*=0.450, **Figure 1C**; DSS, *p*=0.869, **Supplementary Figure S1C**; DFS, *p*=0.409, **Supplementary Figure S1C**; *PMS2*: OS, *p*=0.531, **Figure 1D**; PFS, *p*=0.625, **Figure 1D**; DSS, *p*=0.647, **Supplementary Figure S1D**; DFS, *p*=0.303, **Supplementary Figure S1D**);. In addition, the area under the curve (AUC) of *MSH2* (**Figure 1E**) and *MSH6* (**Figure 1F**) for 1-,3-, and 5-years OS were higher than those of *MLH1* (**Figure 1G**) and *PMS2* (**Figure 1H**). All of these indicate that *MSH2* and *MSH6*, but not *MLH1* and *PMS2*, may be related to the prognosis of EC patients.

### 3.2 Relationship between MMR genes and clinicopathological features of EC and cox regression analysis

**Figure 2:**
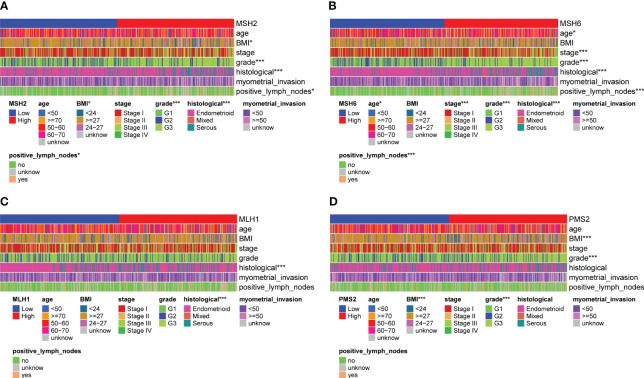
*MSH2* and *MSH6*, but not *MLH1* and *PMS2*, were related to multiple crucial clinicopathological features of EC. The expression of *MSH2* **(A)** and *MSH6* **(B)** was associated with various clinicopathological features, like the age, BMI, FIGO stage and grade, histological and lymph node metastasis of EC patients, while the expression of *MLH1* **(C)** and *PMS2* **(D)** was not relevant to multiple clinicopathological features of EC. *p < 0.05, ***p < 0.001.

By evaluating the relationship between the expression of four MMR genes and the clinicopathological features of EC, we found that *MSH2* (**Figure 2A**) and *MSH6* (**Figure 2B**) were associated with various clinicopathological features. For example, the expression level of *MSH2* (**Supplementary Figure S2A**) was higher in patients with BMI<27, Federation International of Gynecology and Obstetrics (FIGO) grade III (G3), serous carcinoma and lymph node metastasis; The expression level of *MSH6* (**Supplementary Figure S2B**) was higher in patients with age over 60, BMI<27, FIGO stage III, G3, serous carcinoma and lymph node metastasis. These results suggested that patients with high *MSH2* and *MSH6* expression had more aggressive disease features, which were consistent with the worse survival outcomes of patients in the *MSH2* and *MSH6* high expression groups mentioned above. In comparison, the expression of *MLH1* (**Figure 2C**; **Supplementary Figure S2C**) and *PMS2* (**Figure 2D**; **Supplementary Figure S2D**) were not relevant to multiple clinicopathological features of EC.

We conducted Cox regression analysis to verify whether *MSH2* and *MSH6* can be viewed as independent prognostic factors. In univariate Cox analysis, the *p* values of the stage (*p*<0.001), grade (*p*=0.022), histological (*p*=0.006), lymph node metastasis (*p*<0.001) and *MSH6* (*p*=0.008) were significant, but only *MSH6* (*p*=0.005) remained an independent risk factor for predicting adverse outcomes in multivariate Cox regression analysis (**Supplementary Table S1**).

### 3.3 *MSH6* correlated with immune score, immune infiltration, immune checkpoint expression and ICIs Reactivity in EC

**Figure 3:**
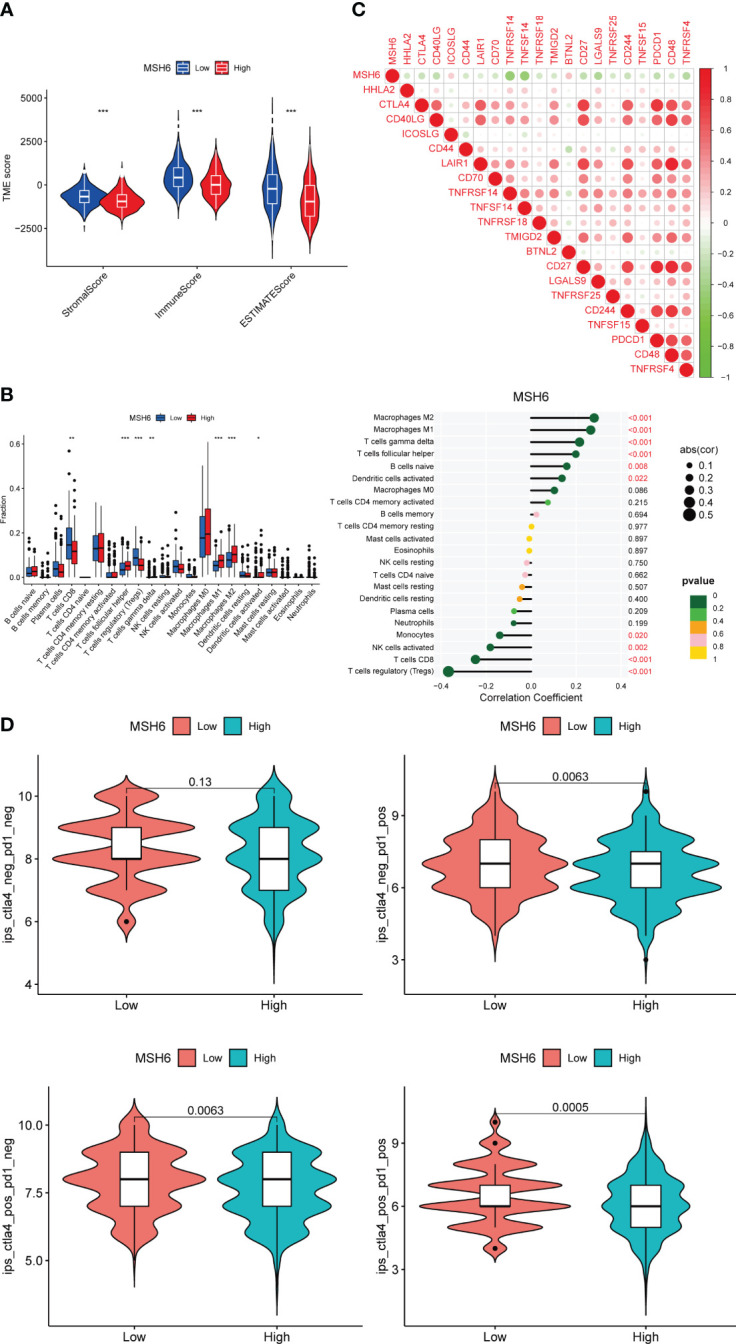
*MSH6* correlated with the immune score, immune infiltration, immune checkpoint expression and ICIs reactivity in EC. The lower the expression level of *MSH6*, the higher the immune, stromal and ESTIMATE scores **(A)**, the higher the infiltration level of multiple immune cells **(B)** and the immune checkpoint expression **(C)**, and the higher the scores of multiple IPS **(D)**. *p < 0.05, **p <0.01, ***p < 0.001.

Given the high immunogenicity of dMMR EC, we tried to analyze the relationship between *MSH6* and the immune score and immune infiltration in EC. The results demonstrated that the immune, stromal and ESTIMATE scores of the low expression group of *MSH6* were significantly higher than those of the high expression group (**Figure 3A**). These suggest that *MSH6* is associated with immune infiltration. We next employed CIBERSORT to analyze the relationship between *MSH6* and 22 types of immune cell infiltration. We found that the expression of *MSH6* (**Figure 3B**) was significantly associated with the infiltration of various immune cells. For example, it was positively correlated with the infiltration of M1 macrophages, M2 macrophages, gamma delta T cells (γδT), follicular helper T cells (Tfh), naïve B cells and activated dendritic cells but negatively correlated with the infiltration of monocytes, activated NK cells, CD8^+^T cells and regulatory T cells (Tregs). We believe that patients in the *MSH6* low expression group have a more active immune status, suggesting that they have a stronger antitumor immune response, which is also consistent with their better prognosis.

Along with immune infiltration, the expression of immune checkpoints such as PD-1 and its ligand (PD-L1) are also associated with the reactivity of dMMR ECs to ICIs. We evaluated the relationship between *MSH6* and immune checkpoint related genes in EC and found that the expression of *MSH6* was negatively correlated with the expression of multiple immune checkpoint related genes, including *CTLA-4* and *PDCD1* (**Figure 3C**).

In conclusion, the negative correlation between *MSH6* and active immune cell infiltration and immune checkpoint expression suggests that the expression of *MSH6* is related to the response to ICIs treatment, the lower its expression, the higher the responsiveness of patients. To verify this hypothesis, we analyzed the relationship between *MSH6* and multiple IPS scores of EC. It was found that IPS-CTLA-4 negative-PD-1 positive (*p*=0.0063), IPS-CTLA-4 positive-PD-1 negative (*p*=0.0063) and IPS-CTLA-4 positive-PD-1 positive (*p*=0.0005) scores were higher in the low expression group of *MSH6* (**Figure 3D**), suggesting that patients in the low expression group had higher reactivity against anti-CTLA-4 antibody and/or anti-PD-1 antibody.

### 3.4 Repeat analysis in *MSH6* wild-type tumor samples

Loss of MMR function is associated with cancer risk, progression and treatment responsiveness. Knijnenburg et al. analyzed the genomic and molecular landscape of DNA damage repair deficiency in pan-cancer using the TCGA database (30). They found that among 33 cancer types, UCEC had the most changes in the DNA damage repair gene somatic alterations, among which the incidence of MMR pathway-related gene mutations, especially *MSH6*, ranks second at 41%. The expression of *MSH6* with loss-of-function mutation differs from that of wild-type *MSH6*.

**Figure 4:**
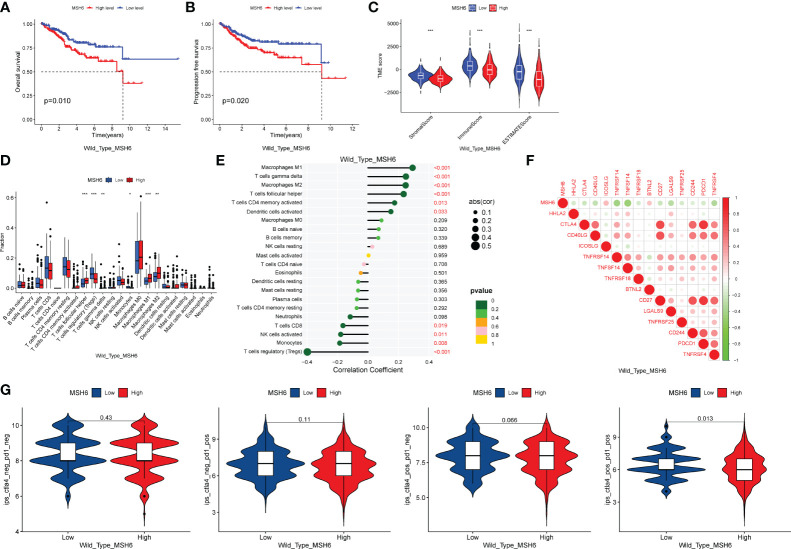
After excluding samples with *MSH6* mutations, *MSH6* was still associated with survival, immune infiltration, immune checkpoint expression and ICIs treatment responsiveness in EC. In *MSH6* wild-type endometrial cancer samples, compared with the high expression group of *MSH6*, the low expression group had higher OS **(A)** and PFS **(B)**, as well as higher immune, stromal and ESTIMATE scores **(C)**; Correlation analysis showed that *MSH6* was negatively correlated with the infiltration of multiple active immune cells **(D, E)** and the expression of immune checkpoints **(F)**; Patients with low *MSH6* expression had better response to anti-CTLA-4 and anti-PD-1 treatment **(G)**. OS, Overall Survival; PFS, Progress Free Survival; EC, endometrial cancer. *p < 0.05, **p <0.01, ***p < 0.001.

To exclude the impact of *MSH6* mutations on our results, we downloaded the single nucleotide polymorphism data of UCEC from TCGA and obtained 432 *MSH6* wild-type tumor samples and 75 *MSH6* mutant tumor samples. We performed all the previously mentioned analyses on the remaining *MSH6* wild-type tumor samples after removing tumor samples with *MSH6* mutations and obtained results consistent with those from before. Compared with the high *MSH6* expression group, the low *MSH6* expression group had higher OS (*p*=0.010, **Figure 4A**) and PFS (*p*=0.020, **Figure 4B**), as well as higher immune, stromal and ESTIMATE scores (**Figure 4C**). Correlation analysis indicated that *MSH6* was negatively correlated with the infiltration of CD8^+^T cells, activated NK cells, monocytes and Tregs, while positively correlated with the infiltration of M1 macrophages, M2 macrophages, etc (**Figures 4D, E**). *MSH6* was also negatively correlated with the expression of multiple immune checkpoint related genes (**Figure 4F**). In addition, the IPS-CTLA-4 positive-PD-1 positive (*p*=0.013) score in the group with low *MSH6* expression was higher than that in the group with high *MSH6* expression, suggesting a better response to anti-CTLA-4 and anti-PD-1 treatment in patients with low *MSH6* expression (**Figure 4G**).

### 3.5 Differences in biological functions between *MSH6* high and low expression groups

**Figure 5:**
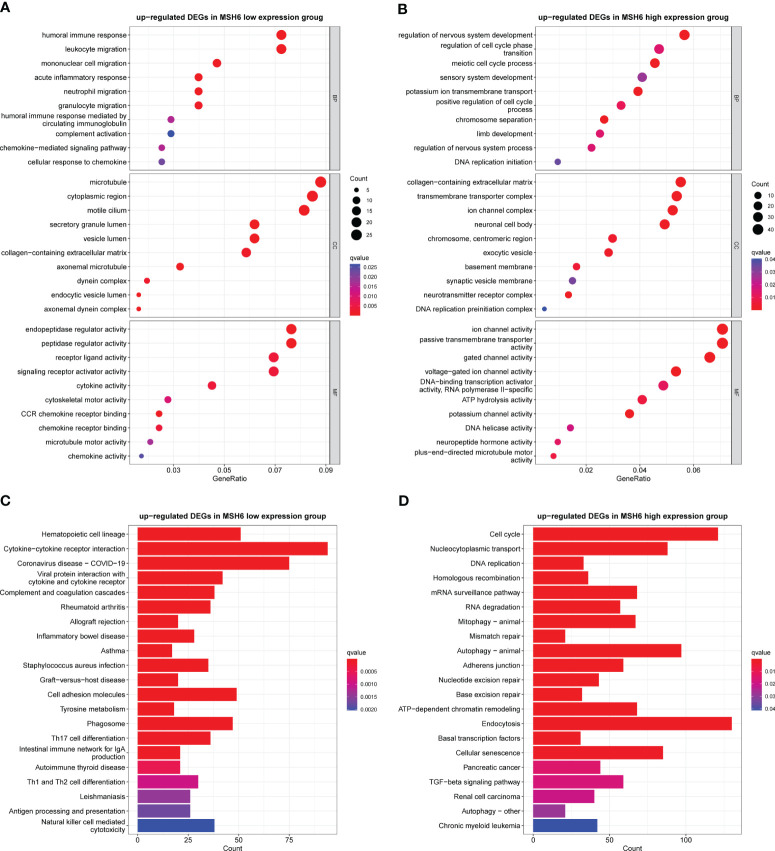
The GO and KEGG enrichment results in the *MSH6* high and low expression groups of endometrial cancer. Representative GO terms **(A)** and KEGG pathways **(C)** enriched in the *MSH6* low expression group mainly regulated immune response. Representative GO terms **(B)** and KEGG pathways **(D)** enriched in the *MSH6* high expression group were related to cell cycle regulation, DNA damage repair and tumorigenesis. GO: gene otology; KEGG: Kyoto Encyclopaedia of Genes and Genomes.

To explore the biological functions and pathways that may be affected by changes in *MSH6* expression in endometrial cancer, we used R to conduct GO and KEGG analyses on the DEGs upregulated in the *MSH6* high and low expression groups, respectively. GO enrichment analysis consisted of three parts: biological process (BP), cellular component (CC), and molecular function (MF). In the *MSH6* low expression group, the enriched BP terms were mainly related to immune cell migration, immune response and chemokine response, the enriched CC terms were primarily associated with the microtubule, motile cilia and dynein complex, and the enriched MF terms were mainly related to various enzyme regulator activity, cytokine activity and motor activity, etc (**Figure 5A**). In addition, the KEGG pathways enriched in the *MSH6* low expression group were involved in multiple immune-related diseases, such as inflammatory bowel disease and asthma, and were also related to T cell differentiation and natural killer cell-mediated cytotoxicity (**Figure 5C**). These results all suggested that genes enriched in the *MSH6* low expression group were mainly involved in regulating immune responses, consistent with the higher immune score (**Figures 3A**, **4C**) and more active immune infiltration (**Figures 3B**, **4D, E**) in the *MSH6* low expression group. In the *MSH6* high expression group, the enriched GO terms were mainly related to the regulation of mitosis, DNA replication and organism development (**Figure 5B**); the enriched KEGG pathways were not only associated with the cell cycle and DNA replication and DNA damage repairs related pathways such as homologous recombination, mismatch repair and base excision repair, it also involved multiple cancer-related pathways such as pancreatic cancer, renal cell carcinoma and chronic myeloid leukemia (**Figure 5D**). These results suggested that genes enriched in the *MSH6* high expression group were mainly related to cell proliferation, and the dysregulation of cell proliferation can lead to the occurrence of cancer, which was consistent with the previously obtained worse survival outcomes of endometrial cancer patients in the high expression group of *MSH6* (**Figures 1B**, **4A, B**).

### 3.6 *MSH6* correlated with the proliferation, migration and invasion ability of endometrial cancer cells

**Figure 6:**
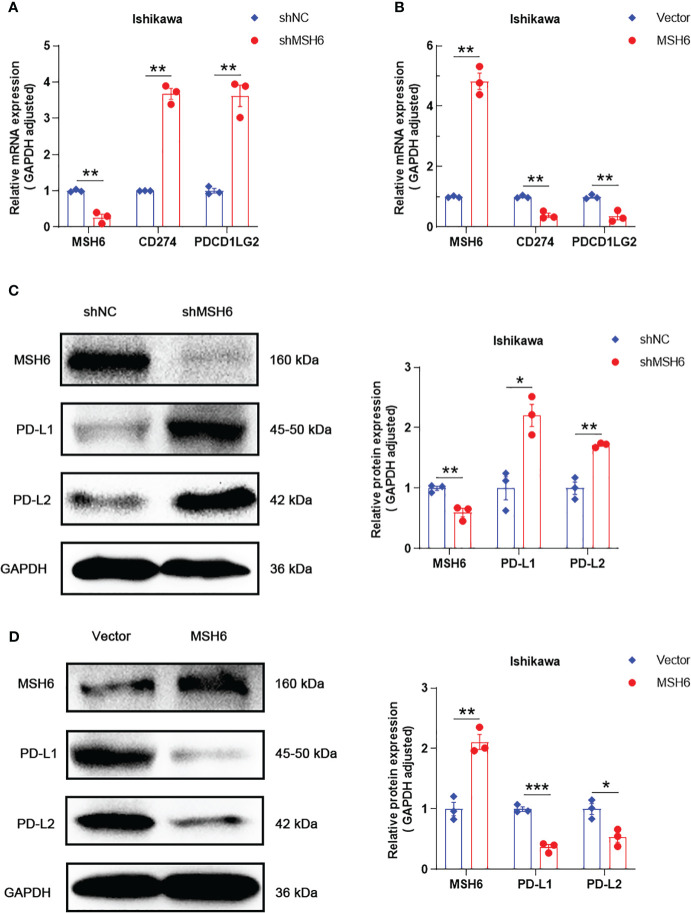
*MSH6* correlated with the expression of PD-L1 and PD-L2 in Ishikawa cells. RT-qPCR and WB confirmed that after knocking down *MSH6* in Ishikawa cells, the expression levels of PD-L1 and PD-L2 were higher than those in the control group **(A, C)**, while the opposite trend was observed after overexpressing *MSH6* **(B, D)**.*p < 0.05, **p <0.01, ***p < 0.001.

**Figure 7:**
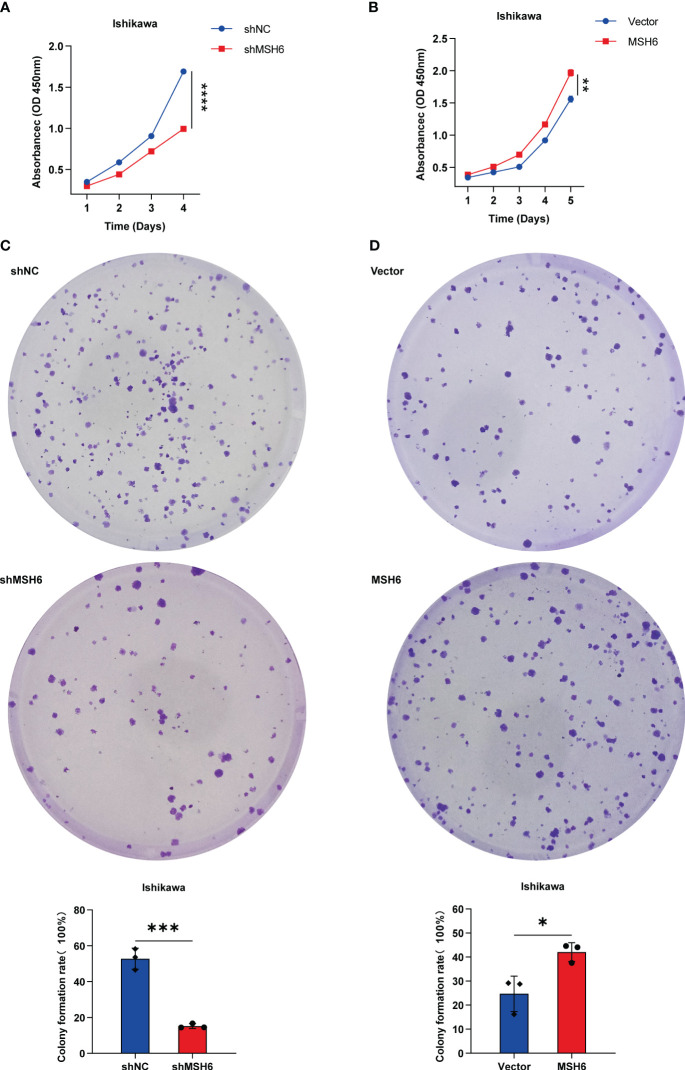
*MSH6* correlated with the proliferation ability of Ishikawa cells. Cell proliferation and clone formation assay confirmed that the proliferation ability of *MSH6* knockdown Ishikawa cells was weaker than that of the control cells **(A, C)**, while in the *MSH6* overexpression Ishikawa cells, it was stronger than that of the control cells **(B, D)**. *p < 0.05, **p <0.01, ***p < 0.001, ****p < 0.0001.

**Figure 8:**
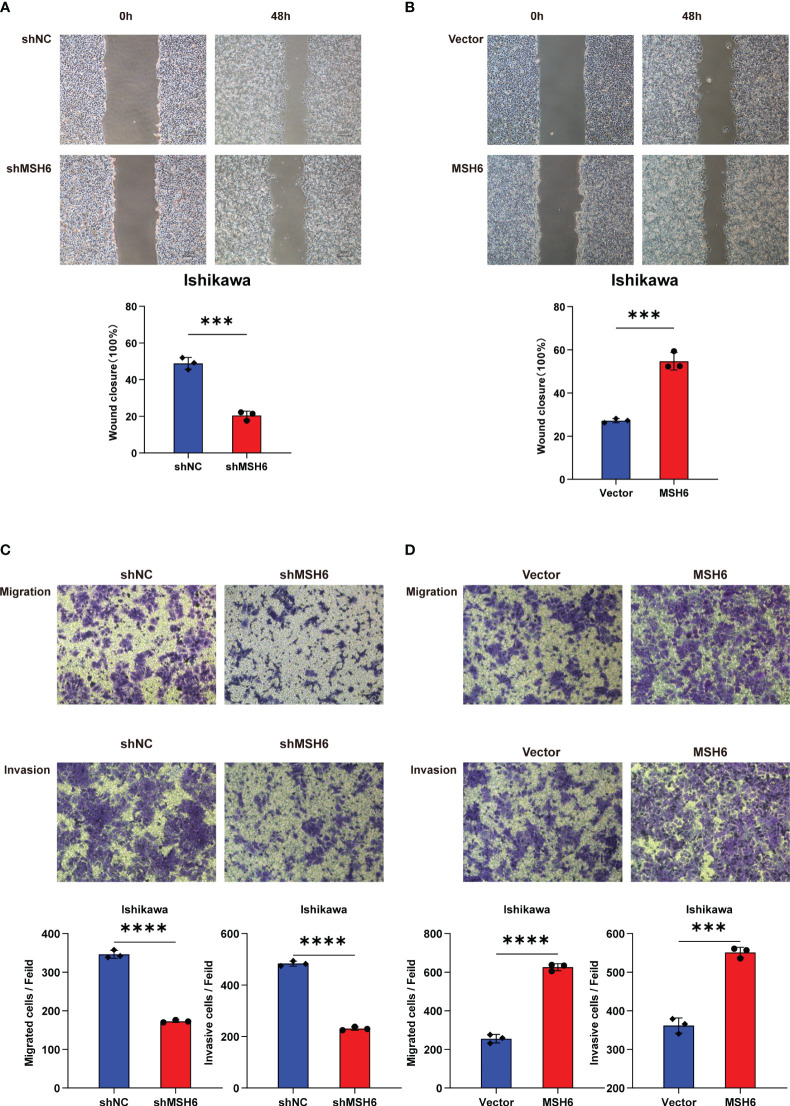
*MSH6* correlated with the migration and invasion ability of Ishikawa cells. Wound healing and Transwell assay confirmed that the migration and invasion ability of *MSH6* knockdown Ishikawa cells was weaker than that of the control cells **(A, C)**, while the opposite trend was observed after overexpressing *MSH6* **(B, D)**. ***p < 0.001, ****p <0.0001.

To verify the results of the aforementioned bioinformatics analysis, we conducted *in vitro* experiments. We infected endometrial cancer cells (Ishikawa and HEC-1B) with lentivirus that can knock down or overexpress *MSH6* and confirmed its infection effect by RT-qPCR and WB (**Figure 6**; **Supplementary Figure S3**). The cell proliferation and clonal formation assay indicated that the proliferation ability of endometrial cancer cells after *MSH6* knockdown was significantly weaker than that of the control group (**Figures 7A, C**; **Supplementary Figures S4A, C**), while it was significantly stronger than that of the control group after overexpression of *MSH6* (**Figures 7B, D**; **Supplementary Figures S4B, D**). The wound healing and Transwell assay revealed that the migration and invasion ability of the *MSH6* knockdown group was significantly inferior to its control group (**Figures 8A, C**; **Supplementary Figures S5A, C**), while in the *MSH6* overexpression group, it was significantly stronger than its control group (**Figures 8B, D**; **Supplementary Figures S5B, D**).

### 3.7 *MSH6* correlated with the expression of PD-L1 and PD-L2

RT-qPCR and WB also confirmed that the expression levels of PD-L1 and PD-L2 in cells after *MSH6* knockdown were significantly higher than those in the control group, while in cells after *MSH6* overexpression, they were significantly lower than those in the control group (**Figure 6**; **Supplementary Figure S3**).

## 4 Discussion

This study found through bioinformatics analysis that *MSH6* was associated with clinicopathological features, prognosis, immune infiltration, immune checkpoint expression and ICIs reactivity in EC. The lower expression level of *MSH6* was associated with less aggressive disease features, higher levels of immune cell infiltration (especially CD8^+^T cells) and immune checkpoint expression in EC patients, which may be related to higher response to ICIs and better prognosis in EC patients. GO and KEGG analyses confirmed that the genes enriched in the *MSH6* low expression group were mainly involved in regulating immune response, while the genes enriched in the *MSH6* high expression group were related to tumorigenesis. *In vitro* experiments also confirmed that the proliferation, migration and invasion ability of cells in the *MSH6* knockdown group were weaker than those in the control group, but the expression levels of PD-L1 and PD-L2 were higher than those in the control group. There was an opposite trend in the overexpression group of *MSH6* and its control group.

It is worth noting that when using CIBERSORT to analyze the relationship between *MSH6* and immune cell infiltration, we found that the trend of Tregs infiltration was consistent with CD8^+^T cells but opposite to M2 macrophages. Previous studies on the immune microenvironment of various tumor types, such as nasopharyngeal cancer (31), colorectal cancer (32), and prostate cancer (33), have suggested that Tregs are positively correlated with M2 macrophages, and when they highly infiltrate tumor tissue, they usually indicate a poor prognosis. Research by Sun (34) and Tiemessen (35) also found that Tregs can promote the differentiation of monocytes into M2 macrophages. These studies typically analyzed all tumor samples uniformly without distinguishing their mismatch repair status. On the other hand, Michel et al. (36) found that the infiltrating levels of CD8^+^T cells and Foxp3^+^Tregs in MSI-H colorectal cancer were significantly higher than those in microsatellite stable (MSS) colorectal cancer and the two cell types were positively correlated. They believed that microsatellite status also affects the density of infiltrating Tregs. Asaka et al. (37) also found that dMMR EC and PD-L1 positive EC had higher levels of CD8^+^T cells, Fxop3^+^Tregs, PD-1^+^ immune cells and PD-L1^+^ immune cells than mismatch repair proficient (pMMR) EC or PD-L1 negative EC, and they suggested that in addition to MMR status, PD-L1 was also associated with T cell-inflamed phenotype. Spranger (38) found that CD8^+^T cells induce the expression of PD-L1 in tumor tissues by secreting IFN-r. On the other hand, CD8^+^T cells can induce *in situ* proliferation of Tregs, which can also recruit Tregs into the tumor by secreting CCL22 and binding with CCR4 on the surface of Tregs. The two mechanisms work together to increase the infiltration level of Tregs in tumors. In conclusion, we speculated that there are three possible reasons for the same trend of Treg infiltration as CD8^+^T cells but opposite to M2 macrophages in our study. One is that MSH6, as a mismatch repair protein, affects the infiltration of Tregs. Second, given the inverse correlation between *MSH6* and CD8^+^T cell infiltration, *MSH6* low-expression tumors have a high degree of CD8^+^T cell infiltration, and these highly infiltrated CD8^+^T cells recruit Tregs to tumors by inducing *in situ* proliferation of Tregs or by secreting cytokines. Third, PD-L1 expression was increased in tumors with low *MSH6* expression, and these highly expressed PD-L1 may also affect Tregs infiltration.

As a responder to DNA damage, the mismatch repair system plays an important role in maintaining genome stability and preventing tumorigenesis. Many studies have found that the overexpression of core genes of the mismatch repair system is related to the occurrence of various tumors. Chen et al. (39) demonstrated that *MSH6* was an overexpressed oncogene in human glioblastoma multiforme tissues that can promote gliomagenesis. In studies about prostate cancer, oral squamous cell carcinoma and melanoma, the overexpression of MMR proteins was associated with poor prognosis (27–29). Lemetre (40) and Berg (41), respectively, used the TCGA database and their own endometrial cancer samples to analyze and found that MSH6 was an independent prognostic marker, and patients with low expression of MSH6 had better survival outcomes. Our results were consistent with their findings. Given the high mutation rate of *MSH6* in endometrial cancer, we not only conducted analysis in unclassified tumor samples but also innovatively verified the results in the remaining *MSH6* wild-type tumor samples after removing those with *MSH6* mutants and obtained consistent results with them. Furthermore, to our knowledge, we are the only study that analyzed the relationship between *MSH6*, immune infiltration and ICIs treatment responsiveness in endometrial cancer. Previous studies primarily compared pMMR tumors with dMMR tumors or between dMMR tumors caused by *MLH1* promoter hypermethylation(*MLH1*-PHM) or MMR-related gene mutation. For example, Kaneko (42) suggested that dMMR ECs with *MLH1*- PHM had a worse prognosis than pMMR ECs and Lynch syndrome (LS) associated dMMR ECs (dMMR ECs without *MLH1*-PHM); Sloan (43) found that the expression of PD-L1 was the highest in LS-associated dMMR ECs, followed by dMMR ECs with *MLH1*-PHM and finally pMMR ECs. Moreover, the MMR immunohistochemical staining pattern most consistent with PD-L1 expression was MSH6 expression loss. They hypothesized which MMR protein defect was the most important variable regulating PD-L1 expression in tumors, regardless of whether it undergoes germline or somatic deficiency. We lack information on the correlation between different involved MMR genes (*MLH1*, *MSH2*, *MSH6*, or *PMS2*) and the prognosis and ICIs reactivity in EC. We supplemented this for the first time and confirmed that *MSH6* is closely associated with prognosis, immune infiltration, immune checkpoint expression and ICIs reactivity in EC. In 2021, by constructing dMLH1 tumor cells and mouse dMLH1 tumor models, Lu et al. (44). demonstrated that deletion of *MLH1* expression improves tumor infiltration of CD8^+^ T cells and enhances ICIs reactivity by promoting cytoplasmic DNA aggregation and activating cGAS- STRING pathway. These also suggested that MMR genes themselves were related to immune infiltration and ICIs reactivity.

The limitation of this study is the lack of our own clinical samples of endometrial cancer to validate the results, especially the need to verify the relationship between *MSH6* and immune infiltration, immune checkpoint expression and ICIs treatment responsiveness. Immune checkpoint inhibitors are not routinely used in patients with endometrial cancer. Only some advanced or relapsed patients with multiple drug resistance choose to receive ICIs treatment. More time is needed to collect sufficient sample size. Understanding the communication between tumor cells and immune cells and their response to ICIs is important to guide drug administration and elucidate the mechanism of drug resistance. Single-cell NRA sequencing (sc-RNA-seq), as a powerful technique, can be used to explore the heterogeneous cellular, molecular characteristics, and intercellular communication within tumors (45). It can also be used to identify key cell types, genes, regulons and pathways with pro-tumor and antitumor potential, guiding us to explore the mechanisms related to response and resistance to ICIs treatment and biomarkers with predictive significance (46). In the future, it is necessary to collect clinical samples of endometrial cancer or construct mouse models to explore the relationship between different MMR genes, especially *MSH6*, and immune infiltration and ICIs response by using sc-RNA-seq or distinguish the cellular and molecular characteristics of different MMR deficiency patterns to find the cause for primary or secondary resistance of dMMR EC to ICIs treatment.

## 5 Conclusions

In conclusion, we confirmed that the expression of *MSH6* was inversely correlated with the prognosis, immune score, immune infiltration, immune checkpoint expression and ICIs response of EC. *MSH6* is anticipated to be a viable biomarker for predicting prognosis, immune status, immune checkpoint expression and perhaps the response to immunotherapy in EC patients. This study also points out new directions for potential drug development. Pharmacists can develop inhibitors that target the MMR genes, especially *MSH6*. These drugs may be used to treat refractory or metastatic advanced MSS/pMMR solid tumors, or they can be combined with ICIs to improve their reactivity in MSI-H/dMMR solid tumors. In addition, given that the infiltration trend of Tregs was similar to that of CD8 ^+^ T cells, we speculated that combining Tregs-targeted therapy and ICIs may improve patient reactivity and prognosis in the presence of a preexisting T cell-inflamed tumor microenvironment.

## Data availability statement

The datasets presented in this study can be found in online repositories. The names of the repository/repositories and accession number(s) can be found in the article/**Supplementary Material**.

## Ethics statement

Ethical approval was not required for the studies on humans in accordance with the local legislation and institutional requirements because only commercially available established cell lines were used. Ethical approval was not required for the studies on animals in accordance with the local legislation and institutional requirements because only commercially available established cell lines were used.

## Author contributions

L-ZZ: Writing – original draft, Conceptualization, Data curation, Formal analysis, Methodology, Software, Validation, Visualization, Writing – review & editing. H-QX: Conceptualization, Methodology, Project administration, Supervision, Writing – review & editing. JC: Conceptualization, Funding acquisition, Project administration, Resources, Supervision, Writing – review & editing.

## Supplementary material

Supplementary Figure 1
*MSH2* and *MSH6*, but not *MLH1* and *PMS2*, related to the DSS and DFS of EC patients. The DSS and DFS curves of the low expression groups of *MSH2* **(A)** and *MSH6* **(B)** were significantly higher than those of their high expression groups, while there was no significant difference between the high and low expression groups of *MLH1* **(C)** and *PMS2* **(D)**. DSS, Disease Specific Survival; DFS, Disease Free Survival; EC, endometrial cancer.

Supplementary Figure 2 Relationship between MMR genes and clinicopathological features of EC. Compared with patients with low expression of *MSH2* and *MSH6*, patients with high expression of *MSH2* and *MSH6* had higher stage and grade, more serous carcinoma, and higher incidence of lymph node metastasis **(A, B)**, while *MLH1* and *PMS2* expression were independent of most these clinicopathological features **(C, D)**.

Supplementary Figure 3 Validation of the relationship between *MSH6* and the expression of PD-L1 and PD-L2 in HEC-1B cells. Verify the knockdown and overexpression efficiency of *MSH6* by RT-qPCR **(A, B)** and WB in HEC-1B cells **(C, D)**, and analyze the mRNA and protein expression of immune checkpoint related genes after knockdown or overexpression of *MSH6*. *p < 0.05, **p <0.01.

Supplementary Figure 4 Validation of the relationship between *MSH6* and cell proliferation in HEC-1B cells. The effect of *MSH6* knockdown **(A, C)** or overexpression **(B, D)** on cell proliferation activity was verified in HEC-1B cells by cell proliferation and clone formation assay. *p < 0.05, **p <0.01.

Supplementary Figure 5 Validation of the relationship between *MSH6* and cell migration and invasion ability in HEC-1B cells. The effect of *MSH6* knockdown **(A, C)** or overexpression **(B, D)** on cell migration and invasion ability was verified in HEC-1B cells by wound healing and Transwell assay. **p < 0.01, ***p <0.001, ****p < 0.0001.
