# Supplementary figures and images for: Mismatch repair gene MSH6 correlates with the prognosis, immune status and immune checkpoint inhibitors response of endometrial cancer
Source: Front Immunol. 2024 Feb 8;15:1302797. doi: 10.3389/fimmu.2024.1302797 (PMC10881679; doi:10.3389/fimmu.2024.1302797)

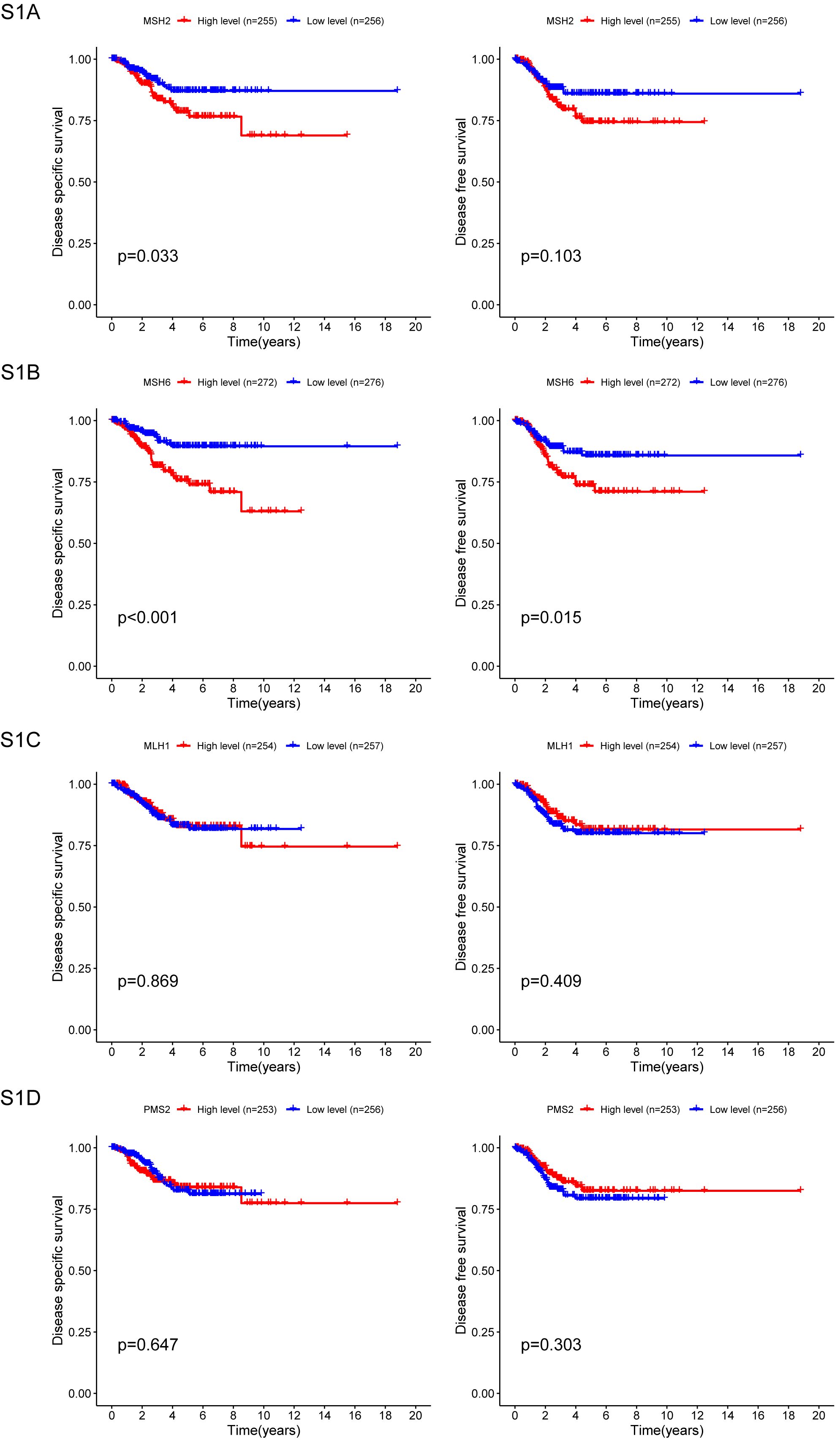

Supplement: Supplementary Figure 1 — MSH2 and MSH6, but not MLH1 and PMS2, related to the DSS and DFS of EC patients. The DSS and DFS curves of the low expression groups of MSH2 (A) and MSH6 (B) were significantly higher than those of their high expression groups, while there was no significant difference between the high and low expression groups of MLH1 (C) and PMS2 (D). DSS, Disease Specific Survival; DFS, Disease Free Survival; EC, endometrial cancer. [file Image_1.tif]

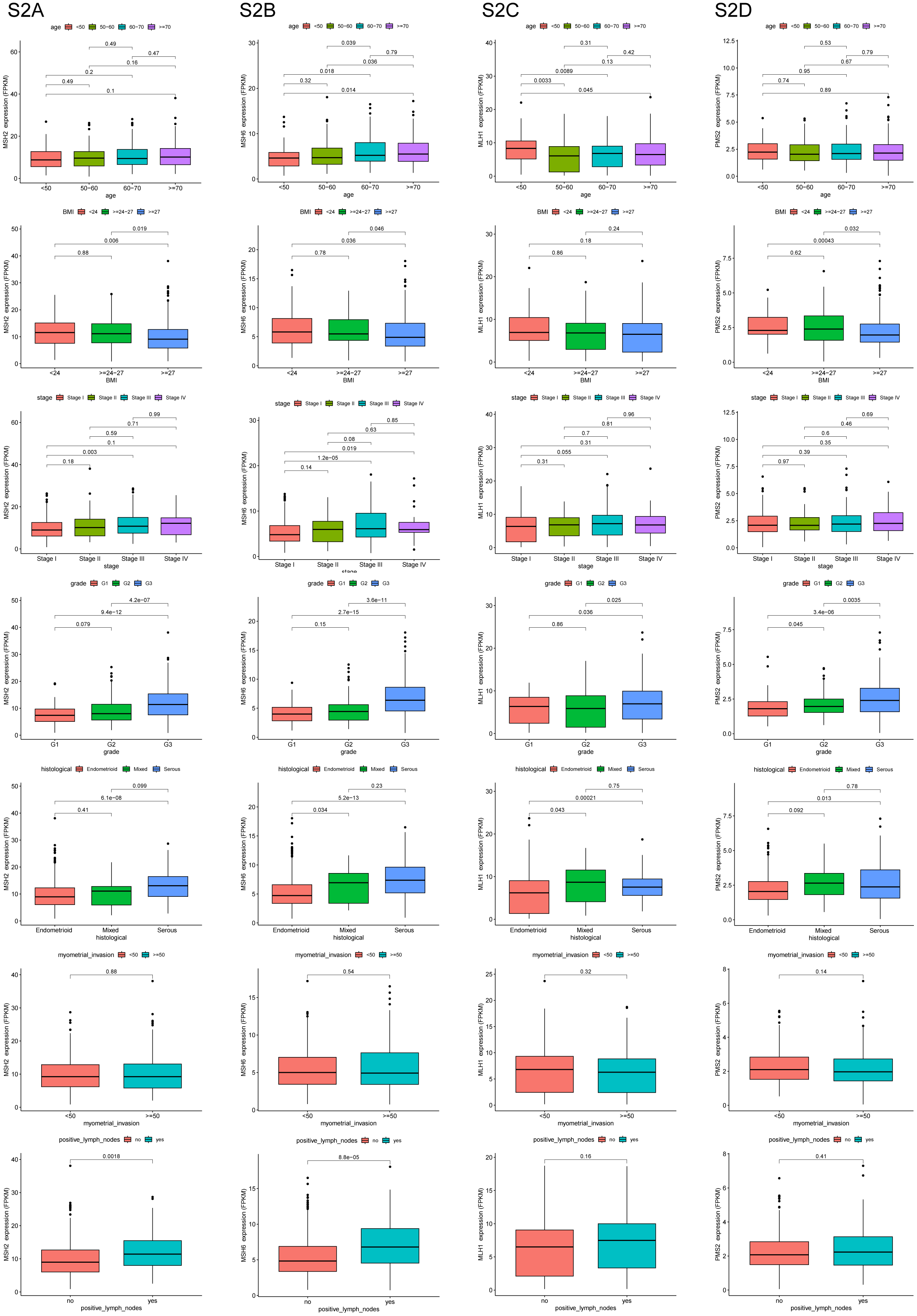

Supplement: Supplementary Figure 2 — Relationship between MMR genes and clinicopathological features of EC. Compared with patients with low expression of MSH2 and MSH6, patients with high expression of MSH2 and MSH6 had higher stage and grade, more serous carcinoma, and higher incidence of lymph node metastasis (A, B), while MLH1 and PMS2 expression were independent of most these clinicopathological features (C, D). [file Image_2.tif]

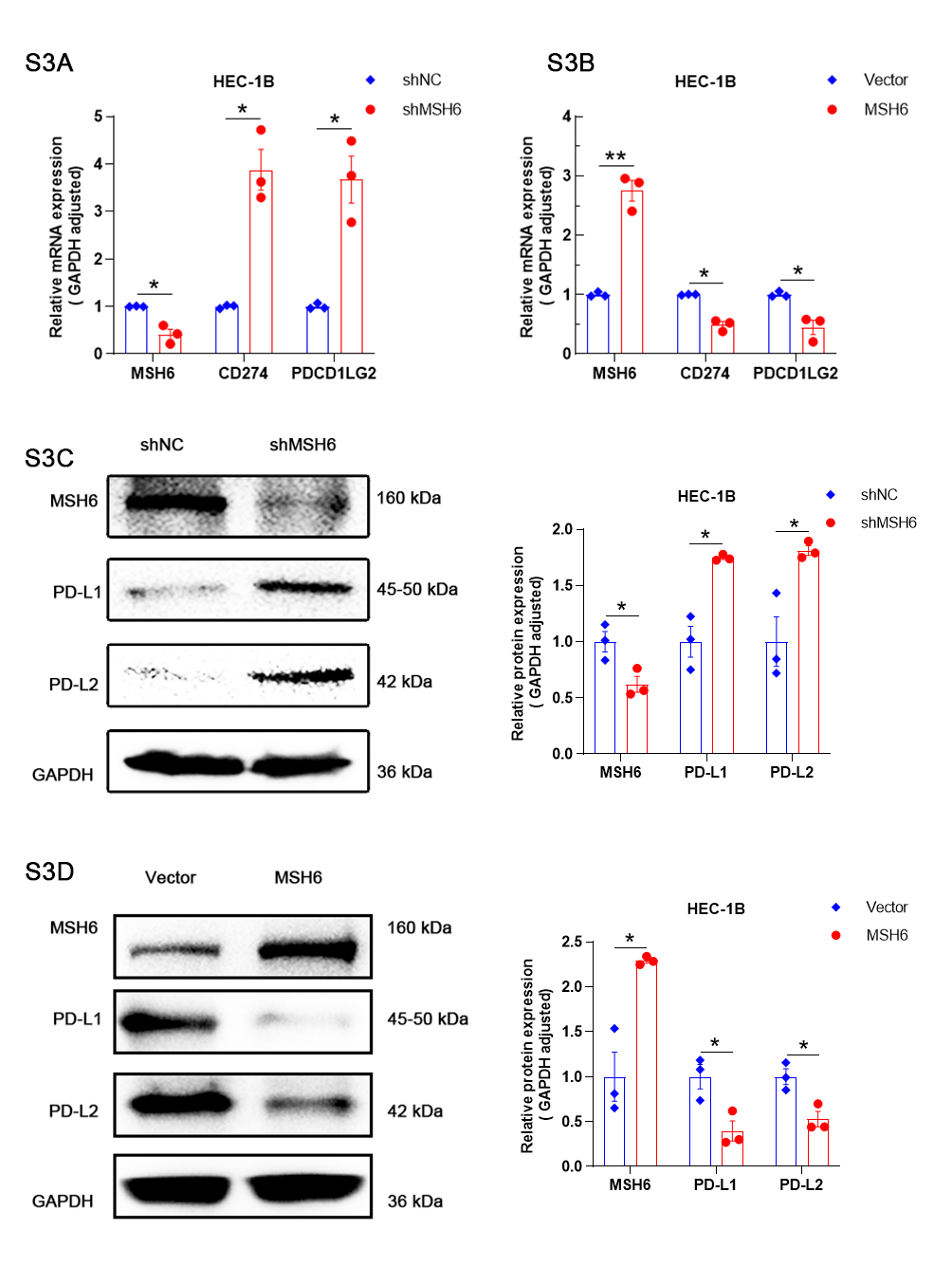

Supplement: Supplementary Figure 3 — Validation of the relationship between MSH6 and the expression of PD-L1 and PD-L2 in HEC-1B cells. Verify the knockdown and overexpression efficiency of MSH6 by RT-qPCR (A, B) and WB in HEC-1B cells (C, D), and analyze the mRNA and protein expression of immune checkpoint related genes after knockdown or overexpression of MSH6. *p < 0.05, **p <0.01. [file Image_3.tif]

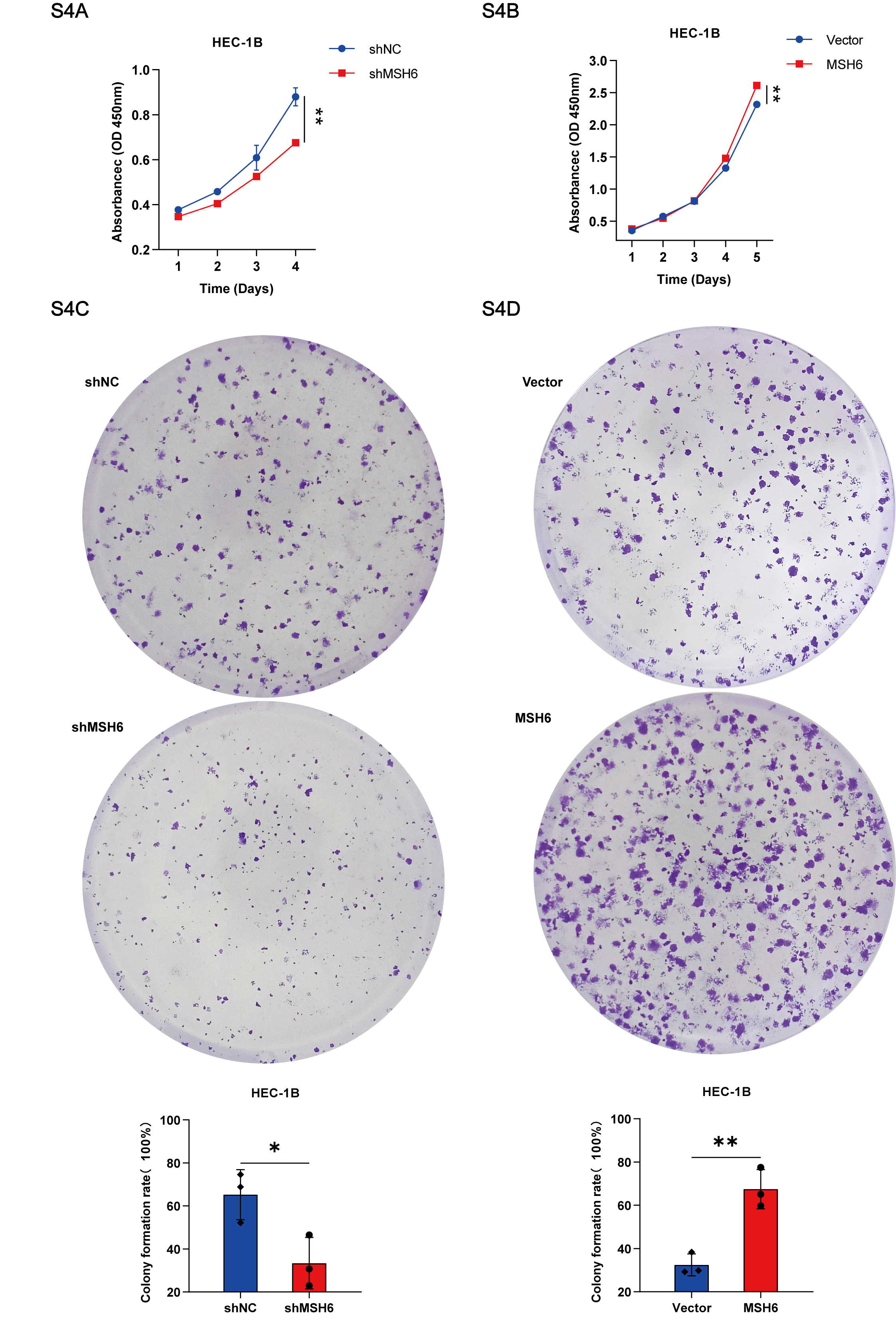

Supplement: Supplementary Figure 4 — Validation of the relationship between MSH6 and cell proliferation in HEC-1B cells. The effect of MSH6 knockdown (A, C) or overexpression (B, D) on cell proliferation activity was verified in HEC-1B cells by cell proliferation and clone formation assay. *p < 0.05, **p <0.01. [file Image_4.tif]

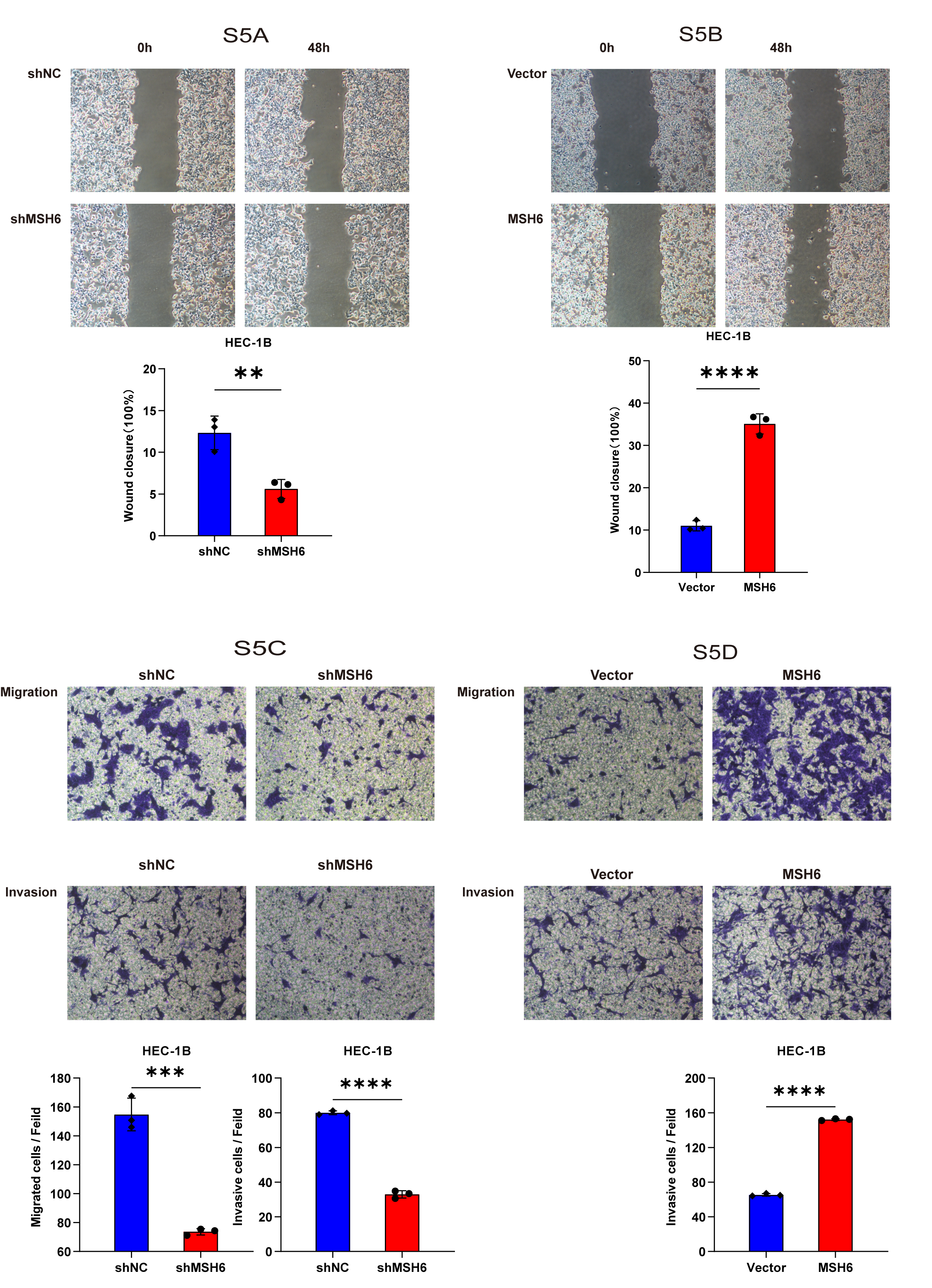

Supplement: Supplementary Figure 5 — Validation of the relationship between MSH6 and cell migration and invasion ability in HEC-1B cells. The effect of MSH6 knockdown (A, C) or overexpression (B, D) on cell migration and invasion ability was verified in HEC-1B cells by wound healing and Transwell assay. **p < 0.01, ***p <0.001, ****p < 0.0001. [file Image_5.tif]
