# Supplementary material for: Mismatch repair gene MSH6 correlates with the prognosis, immune status and immune checkpoint inhibitors response of endometrial cancer
Source: Front Immunol. 2024 Feb 8;15:1302797. doi: 10.3389/fimmu.2024.1302797 (PMC10881679; doi:10.3389/fimmu.2024.1302797)
Supplement: Supplementary file 6 [file Table_1.docx]

**Table S1.** Univariate and multivariate Cox regression analysis of the correlation of MMR genes with OS among EC patients.

| **Parameter** | **Univariate Analysis** | | | **Multivariate Analysis** | | |
| --- | --- | --- | --- | --- | --- | --- |
|  | **HR** | **95% CI** | **P-value** | **HR** | **95% CI** | **P-value** |
| Age | 1.009 | 0.979-1.040 | 0.551 | 1.026 | 0.985-1.069 | 0.216 |
| BMI | 1.017 | 0.978-1.056 | 0.400 | 1.031 | 0.986-1.078 | 0.176 |
| Stage | 1.973 | 1.393-2.794 | <0.001 | 1.384 | 0.746-2.567 | 0.302 |
| Grade | 1.667 | 1.078-2.579 | 0.022 | 1.350 | 0.805-2.263 | 0.255 |
| Histological | 1.605 | 1.143-2.254 | 0.006 | 1.175 | 0.746-1.853 | 0.486 |
| Myometrial invasion | 1.622 | 0.862-3.054 | 0.134 | 0.915 | 0.438-1.910 | 0.812 |
| Positive lymph nodes | 4.928 | 2.537-9.572 | <0.001 | 2.385 | 0.770-7.738 | 0.132 |
| *MLH1* | 0.977 | 0.903-1.056 | 0.553 | 0.951 | 0.785-1.035 | 0.244 |
| *MSH2* | 1.013 | 0.963-1.067 | 0.610 | 0.902 | 0.809-1.006 | 0.064 |
| *MSH6* | 1.145 | 1.036-1.266 | 0.008 | 1.309 | 1.083-1.582 | 0.005 |
| *PMS2* | 0.778 | 0.555-1.089 | 0.144 | 0.738 | 0.484-1.124 | 0.157 |
